# Identification and Isolation of Two Different Subpopulations Within African Swine Fever Virus Arm/07 Stock

**DOI:** 10.3390/vaccines8040625

**Published:** 2020-10-25

**Authors:** Daniel Pérez-Núñez, Eva Castillo-Rosa, Gonzalo Vigara-Astillero, Raquel García-Belmonte, Carmina Gallardo, Yolanda Revilla

**Affiliations:** 1 Centro de Biología Molecular Severo Ochoa, CSIC-UAM, Microbes in Health and Welfare Department, c/ Nicolás Cabrera, 1, 28049 Madrid, Spain; daniel_perez@cbm.csic.es (D.P.-N.); gvigara@cbm.csic.es (G.V.-A.); raquel.g.b@cbm.csic.es (R.G.-B.); 2 Centro de Biología Molecular Severo Ochoa, CSIC-UAM, Genomics and NGS Core Facility, c/ Nicolás Cabrera, 1, 28049 Madrid, Spain; ecastillo@cbm.csic.es; 3 European Union Reference Laboratory for African Swine Fever (EURL), INIA-CISA, Valdeolmos, 28049 Madrid, Spain; gallardo@inia.es

**Keywords:** ASFV, LAVs, Arm/07 stock, Arm/07/CBM/c2, Arm/07/CBM/c4, viral DNA purification, NGS, coverage, CD2v, hemadsorption

## Abstract

No efficient vaccines exist against African swine fever virus (ASFV), which causes a serious disease in wild boars and domestic pigs that produces great industrial and ecological concerns worldwide. An extensive genetic characterization of the original ASFV stocks used to produce live attenuated vaccine (LAV) prototypes is needed for vaccine biosecurity and control. Here, we sequenced for the first time the Arm/07 stock which was obtained from an infected pig during the Armenia outbreak in 2007, using an improved viral dsDNA purification method together with high coverage analysis. There was unexpected viral heterogeneity within the stock, with two genetically distinct ASFV subpopulations. The first, represented by the Arm/07/CBM/c2 clone, displayed high sequence identity to the updated genotype II Georgia 2007/1, whereas the second (exemplified by clone Arm/07/CBM/c4) displayed a hemadsorbing phenotype and grouped within genotype I based on a central region conserved among all members of this group. Intriguingly, Arm/07/CBM/c4 contained a unique EP402R sequence, produced by a single mutation in the N-terminal region. Importantly, Arm/07/CBM/c4 showed in vitro features of attenuated strains regarding innate immune response pathway. Both Arm/07/CBM/c2 and c4 represent well-characterized viral clones, useful for different molecular and virus-host interaction studies, including virulence studies and vaccine development.

## 1. Introduction

African swine fever virus (ASFV), the only member of the family *Asfarviridae* [1], contains a linear, double-stranded DNA molecule genome that ranges in length from about 170 to 194 kbp, showing inverted terminal repeats (ITRs) and covalently closed ends [2,3,4]. The viral genome includes a conserved central region (CCR) and two variable ends, which results in some variation in size among strains [2,3,4,5]. The first ASFV whole genome sequence [6] was completed by the Sanger method on the cell culture-adapted Ba71V strain. Since then, several technologies have been implemented for the sequencing of ASFV populations, including Roche 454 GS FLX, Illumina HiScanSQ, MiSeq, HiSeq, NextSeq500 and Nanopore [5,7,8,9,10,11,12,13,14,15]. However, these technologies depend on high-quality parameters of sequence data to assess the reliability of the results. In fact, low coverage may lead to misinterpretation of the data and underestimation of the variant frequency. Many parameters, in particular mean coverage, also deal with efficiency of viral DNA purification and quality of the sample. An important source of the low coverage seen in next-generation sequencing (NGS) of ASFV genomes is mainly related to the problem of eukaryotic DNA contamination, which has been approached by different strategies including animal infections, purification from animal blood, non-specific DNA amplification or probe-mediated viral DNA enrichment designed with known ASFV-sequences [10,12,15,16].

ASFV is the etiological agent of African swine fever [17,18,19], a serious disease affecting both wild boar and domestic pigs, which lastly emerged from East Africa to the Caucasus in 2007, where it spread to affect 28 countries in Europe, Oceania and Asia, including China [20,21]. The virus is now endemic in China and is currently affecting neighboring countries such as Vietnam, Laos, Myanmar, Korea and the Philippines. The situation is economically dramatic, unbalancing the food chain and representing one of the most important social and industrial animal health concerns worldwide. The rapid spread of the disease proved eradication and regionalization measures to be insufficient for control of the current epidemic.

The development of effective vaccines is currently being attempted by several labs [22,23,24] and indeed is urgently required. Among the different vaccine strategies, one of the most realistic option is the generation of live attenuated vaccines (LAVs), which are usually based either on naturally attenuated ASFV strains or on genetic manipulation of virulent strains in order to experimentally attenuate virulence [25,26,27,28]. An exhaustive genetic characterization using cutting-edge technologies must be performed, both on the parental stocks and the final LAV prototypes. Vaccine prototypes must be characterized not only to confirm the introduced genetic modifications (mostly deletion of specific genes) [28] but extended to the whole genome [25,27]. This approach allows the identification of several genetic factors, such as off-target modifications, parental contamination and the presence of possible subpopulations and/or minor viral variants in the stocks, which eventually could also play a role in the overall safety of LAVs.

Here, we describe for the first time an improved viral DNA purification technique from extracellular viral particles and high-coverage NGS analysis for the genomic characterization of the Arm/07 stock. The Arm/07 stock presented at least two distinct sub-populations: Arm/07/CBM/c2, grouped within genotype II with 99.992% sequence identity to the updated Georgia 2007/1 reference strain [12] and the clone Arm/07/CBM/c4, which showed a high level of heterogeneity within left and right genome ends and presented identity with genotype I strains, ranging from 99.605% (BA71V), 99.503% (E75) to 96.542% (Mzuki_1979). Remarkably, a single deletion of Arm/07/CBM/c4 at 75,213 position within the EP402R gene produced a frameshift that shortened the N-terminal region of this protein compared to other genotype I virulent strains. Therefore, the overall sequence of EP402R from Arm/07/CBM/c4 differed from both virulent and attenuated genotype I strains and from genotype II strains, resulting in a unique EP402R sequence. Furthermore, Arm/07/CBM/c4 showed impaired ability to control cGAS-STING pathway in vitro, similar to NH/P68 attenuated strain, while Arm/07/CBM/c2 prevented STING and IRF3 activation.

## 2. Materials and Methods

### 2.1. Cells and Viruses

The hemadsorbing ASFV isolate Arm/07 (genotype II) was obtained from an epizootic of domestic pigs in Armenia in 2007. The isolate was propagated in three passages in porcine blood monocytes (PBMs) according to the OIE Manual (2019). The low virulent, non-hemadsorbing ASFV NH/P68 (genotype I) isolated in Portugal was obtained in COS-1 cells. Viruses were grown in porcine alveolar macrophages (PAMs) or porcine blood monocytes (PBMs), in DMEM (Dulbecco Modified Eagle Medium) supplemented with 10% pig serum (Sigma, St. Louis, MO, USA) as previously described [29]. African green monkey kidney cells (COS-1) obtained from the American Type Culture Collection (CLR-1650 ATCC), were cultured in DMEM with 5% FBS. Cells were grown at 37 °C in a 5% CO_2_ atmosphere saturated with water vapor in culture medium supplemented with 2 mM L-glutamine, 100 U/mL gentamycin and 0.4 mM nonessential amino acids.

### 2.2. Viral DNA Extraction for NGS Analysis

The Arm/07 virus stock and the selected Arm/07 clones were grown in six P100 dishes of PAMs, with supernatants collected at 3 days post-infection and centrifuged at 8281× *g* o/n at 4 °C. Pellets were resuspended in cold, filtered 10 mM Tris-HCl (pH 8.8), then treated with 0.25 U/µL DNAse I (Sigma), 0.25 U/µL Nuclease S7 (Sigma) and 20 µg/mL RNAse A (Promega) in 800 mM Tris-HCl (pH 7.5), 200 mM NaCl, 20 mM CaCl_2_ and 120 mM MgCl_2_ for 2 h at 37 °C and further incubation with 12 mM EDTA (Sigma) and 2 mM EGTA (Sigma) for 10 min at 75 °C. After that, the solution was treated with 200 µg/mL proteinase K (Sigma) in 0.5% SDS for 1 h at 45 °C, then viral DNA was precipitated by incubating 1:1 with phenol:chloroform:isoamyl alcohol (25:24:1). After centrifugation at 9400× *g* for 3 min at RT, the aqueous fraction was transferred and further incubated with 0.1 volume of 3 M acetic acid (pH5.2), 1 µL LPA (Sigma) and 2 volumes of cold 100% ethanol for 1 h at −80 °C. After centrifugation at 15,890× *g* for 30 min at 4 °C, supernatants were discarded and pellets were washed once with cold 70% ethanol and dried on air before finally being resuspended in 10 mM Tris (pH 8.8).

### 2.3. Isolation of Viral Clones from Arm/07 Stock by Plaque Purification

For the isolation of independent clones from Arm/07 stock, we plated approximately 10^3^ viruses per well of 6-well plates in COS-1 cells. After adsorption for 1.5 h, carboxymethyl cellulose (CMC) with 2% FBS DMEM was added. After 7 days, the appearance of lysis plaques was identified by optical microscope and collected by sterile tips in 40 µL of DMEM and conserved at −80 °C. After three freeze/thaw cycles, extracted virus were used to infect new COS-1 cells with the same procedure explained above. After three rounds of purification, individual clones were amplified and grown in PAMs.

### 2.4. Hemoadsorption (HAD) Assay

PAM (Porcine Alveolar Macrophages) cells were seeded in 12-well plates and mock-infected or infected with either Arm/07/CBM/c2 and Arm/07/CBM/c4 clones at MOI 0.5 in DMEM with 10% pig serum. After 16 hpi, a solution of fresh pig erythrocytes (2 µL of pig erythrocyte sediment per mL of 10% pig serum DMEM) was added to every well. 24 h after pig erythrocytes addition, rosettes were observed under Leica DM IL LED microscope coupled to a Leica DFC3000G camera (Leica Microsystem, Wetzlar, Germany).

### 2.5. Western Blot Analysis

PAM cells were cultured as indicated and mock-infected or infected with either Arm/07/CBM/c2 or Arm/07/CBM/c4 (or NH/P68) at MOI 0.5. Infected cells were collected at 16 hpi, washed with PBS and lysed using radioimmunoprecipitation assay (RIPA) buffer supplemented with protease and phosphatase inhibitors (Roche, Basel, Switzerland). Samples were kept at 4 °C for 30 min, sonicated and centrifuged for 10 min at 15,890× *g* at 4 °C. Supernatants were collected and quantified using BCA assay and 20 µg of each sample Samples were resolved by sodium dodecyl sulfate polyacrylamide gel electrophoresis (SDS-PAGE) and transferred to Immobilon-P membranes (Merck Millipore, Burlington, MA, USA). The membranes were incubated with the following specific primary antibodies: anti-P72 (1:2000, generated in CBMSO), anti-CD2v (1:2000, generated in CBMSO), anti-P32 (S-1D8) (1:6000, kindly provided by S.-Y. Sunwoo), anti-p-STING (Ser366) rabbit monoclonal antibody from Cell Signaling (1/1000), anti-p-IRF3 (Ser396) (4D4G) rabbit monoclonal antibody from Cell Signaling (1/1000) and anti-actin (1:1000, Santa Cruz Biotechnology sc-47778, Dallax, TX, EEUU) diluted in Tris-buffered saline (TBS) supplemented with 1% milk. Membranes were washed three times with TBS and exposed 1 h to specific peroxidase-conjugated secondary antibodies: anti-rabbit and anti-mouse immunoglobulin G coupled to peroxidase (1/5000 and 1/2000, respectively) from Amersham Biosciences (Little Chalfont, UK). and anti-m-IgGκ secondary antibody (1/1000) from Santa Cruz Biotechnology. Chemiluminescence detection was performed using ECL Prime (Amersham Biosciences, Little Chalfont, UK).

### 2.6. Arm/07/CBM/c2 and Arm/07/CBM/c4 Growth Curves

To elucidate the difference in behavior between both Arm/07 clones, the growth rate of each individual clone was calculated. Two 12-well plates were seeded with PAM at a density of 1.6 × 10^6^ PAMs per well and infected with either Arm/07/CBM/c2 or Arm/07/CBM/c4 at MOI = 0.1. After 2 h of adsorption at 37 °C, the viral inoculum was discarded and cells were washed two times with PBS. DMEM containing 10% pig serum was added and cells were incubated for 0, 24, 48 and 72 h post-infection (hpi) at 37 °C and 5% CO_2_.

At each time point, cells were collected and centrifuged 5 min at 250× *g* at room temperature. 100 µL of the supernatant was stored as extracellular virus (EV) and the rest of the supernatant was homogenized with the pellet, submitted to three freeze/thaw cycles and centrifuged 5 min at full speed to precipitate cellular debris. The supernatant was stored as total virus (TV) fraction.

For viral titration, we performed hemadsorption (HAD) assay. Individual 60-well microtest plaques (Greiner) were seeded with PAMs, using a concentration of 10^6^ PAMs per ml DMEM with 10% pig serum using 1 mL for each plate. For infection, 5 µL of the corresponding viral dilution were added. Cells were infected for 16 h and then a solution of fresh pig erythrocytes (2 µL of pig erythrocyte sediment per ml of 10% pig serum DMEM media) was added to every well. Hemadsorption was assessed 96 h after the addition of erythrocytes and the resulting growth curve was plotted using GraphPad prism software.

### 2.7. Nanopore and Illumina Sequencing and Data Analysis

High-quality genomic DNA (100 ng) was submitted to MicrobesNG (Birmingham, UK). Illumina libraries were prepared with an NEBNext Ultra DNA Library Prep Kit (New England Biolabs). DNA samples were fragmented in a Covaris instrument and sequenced on an Illumina MiSeq device as paired-end (2 × 250 bp) reads. Illumina reads were trimmed using Trimmomatic [30] (v0.39). Long reads were sequenced in a GridION instrument from Oxford Nanopore Technologies.

The quality analysis of short and long reads was performed with FastQC [31] (v0.11.8).

For variant calling analysis, the BWA-MEM tool (Burrows-Wheeler Alignment using MEM algorithm) [32] was used to align Illumina reads against the Georgia 2007/1 reference sequence (accession number LR743116.1). Estimated average coverage was calculated based on the percentage of mapped reads and genome size. To separate the two putative subpopulations, bamsplit.py (https://github.com/luntergroup/bamsplit) was used, a Python 3 tool for splitting a BAM file by reads supporting different haplotypes present in a VCF (Variant Call Format) file.

Picard Tools (https://broadinstitute.github.io/picard/) was used to remove read duplicates and the pre-processed alignment files were then used for the variant calling process with GATK [33] (v4.1.2). Numbers of SNPs (Single Nucleotide Polymorphism) and indels were determined and characterized by their location in coding and non-coding regions, as well as by synonymous or nonsynonymous SNPs. Due to the low read mapping quality at the genome ends, variants within the ITR (Inverted Terminal Repeat) regions were not included in the analysis. Minor genetic variants were detected using VarScan [34] (v2.3.9), setting a minimum variant allele frequency above 0.02. Genetic variants were annotated using SnpEff [35] software (v4.3t). For the allele balance distribution plot along the genome, the R package vcfR [36] was employed.

*De-novo* assembly of Illumina reads was generated with SPAdes [37] (v3.14.0) and the contigs obtained were compared with the Georgia 2007/1 reference using BLASTn (v2.9.0+) in the command line. After filtering the assembly, contigs were extended and scaffolded with SSPACE-standard [38] software. To polish the assembly, reads were mapped to the new extended contigs and manually curated to obtain a single contig of 190,145 bp.

The assembly of Arm/07/CBM/c4 genome was performed following the ONT (Oxford Nanopore Technologies, Oxford, UK) assembly and Illumina polishing pipeline by Oxford Nanopore Technologies (available in https://github.com/nanoporetech/ont-assembly-polish). First, Nanopore reads were assembled using Canu [39] (v1.9). The contigs obtained were polished with Racon software [40] (v1.4.16). Illumina reads were mapped onto the polished contigs using BWA-mem and were then used for the correction of the assembly with Pilon (v1.20) [41]. Nanopore reads were aligned against the final genome using Minimap2 tool (v2.11-r797) [42].

The genome assemblies were annotated using PROKKA [43] (v1.13), a tool to annotate prokaryotic genomes and GATU [44], a genome annotation transfer tool based on a closely related organism.

In order to identify differences between genotype II and I strains and Arm/07/CBM/c2 or Arm/07/CBM/c4 genomes, NCBI (National Center for Biotechnology Information) BLASTn with standard parameters and Nucmer [45] (v 4.0.0beta2) were used. Nucmer application was used to discover genetic variants. The identification of modified CDS (Coding Sequence) was accomplished with SnpEff software, used to annotate genetic variants and a subsequent manual identification using IGV viewer [46].

To analyze the differences in EP402R, MGF-110-11L, MGF-110-14L and ASFV_G_ACD_00350 genes between different strains, gene sequences were obtained from NCBI database for each published strain. These sequences were aligned using ClustalW software [47]. Alignment were visualized using Snapgene software (from Insightful Science).

### 2.8. Phylogenetic Analysis

ASFV sequences corresponding to the central conserved region (comprising 129 kb between A224L and I196L genes) from 18 genotype II strains, 23 ASFV sequences corresponding to genotype I strains and 2 ASFV sequences corresponding to genotype X strains (sequences were downloaded from the NCBI database. A multi-fasta file including all 43 sequences downloaded plus Arm/07/CBM/c2 and Arm/07/CBM/c4 genomic sequences was generated and aligned using MAFFT software (v.7.390) with automatic settings.

The generated alignment file was used to build a phylogenetic tree of all downloaded ASFV strains with IqTree software using maximum-likelihood method [48]. We used standard parameters, which identified the most suitable model based on the alignment and ultra-fast bootstrapping of 10,000 times. The most suitable model found by IqTree software was K3Pu+F+R2 (three substitution types model and equal base frequencies + empirical base frequencies + free rate model 2). The tree was rooted using Archaeopteryx, exported to Newick format and modified using Dendroscope software (University of Tübingen, Tübingen, Germany).

For the recombination analysis, the alignment file generated with MAFFT was used. The Recombination Detection Program (RDP, v Beta 5.05) [49] was employed to detect potential recombination events. The default parameters of six methods (RDP, GENECONV, Bootscan, Maxchi, Chimaera, SiSscan) were used and only those events supported by a *p*-value below 0.05 found by at least two methods were reported (see Supplementary Table S2).

### 2.9. PCR and Sanger Sequencing

Conventional PCR for ITR-containing contig placement in Arm/07/CBM/c2 assembled genome at both ends and EP402R indel verification in Arm/07/CBM/c4 assembled genome was performed with Phusion High-Fidelity PCR Master Mix with HF Buffer (Thermo Sicentic, USA) and the following primers: 5′-AAACTTTCATATTGGTAACTTGTTC-3′ and 5′-TATTCGCACTAAAGTGCTATGTTAC-3′; 5′- AGTGAAGATCTATAGCTACGCCTTA-3′ and 5′-TATTCGCACTAAAGTGCTATGTTAC-3′; and 5′-AGCGCGAATTCGCCACCATGATAATAATAGTTATTTTTTTAATGTG-3′ and 5′-ATATGTTCTATTAAATATTTCTGTATTGTTAGG-3′ respectively. Corresponding bands (618 bp, 541 bp and 293 bp respectively) were purified from a 1% agarose gel with Speedtools PCR Clean-Up kit (Biotools, Madrid, Spain). The purified products were subsequently sequenced by the Sanger method (Macrogen, Seoul, Korea).

### 2.10. Data Availability

Read files and assembled genome Arm/07/CBM/c2 together with the annotation have been deposited in the European Nucleotide Archive (ENA) under the study accession number PRJEB38146. Raw reads from clones 1 and 3 have been deposited under the study accession PRJEB40011. Read files and assembled genome Arm/07/CBM/c4 together with the annotation are available under the study accession PRJEB40012.

## 3. Results

### 3.1. Improvements in Viral DNA Purification Allowed Detection of at Least Two Distinct Populations Within the Arm/07 Stock

In order to characterize the ASFV Arm/07 stock, Illumina reads obtained from infected cell lysates were aligned against the ASFV Georgia 2007/1 reference sequence (accession no. LR743116), which is the both geographically and temporally the closest ASFV strain to Arm/07. The initial data obtained, despite the high number of total reads, showed only 2.89% aligned to viral sequences (Table 1). Apart from this, the mean coverage of reads corresponding to the Arm/07 strain was also very low. Eukaryotic DNA contamination was found in the analyzed sample, thus explaining the low number of viral reads.

**Table 1:** Illumina sequencing statistics of African swine fever virus (ASFV) Arm/07 from viral DNA obtained from either intracellular or extracellular virions.

Sample	Total Reads	Aligned Reads	% Aligned Reads	Mean Coverage
Arm/07 (DNA from intracellular virions)	196,190	5675	2.89	6
Arm/07 (DNA from extracellular virions)	357,562	303,524	84.89	338

To overcome cellular DNA contamination and increase the mean sequence coverage, Arm/07 DNA was purified from PAM infection supernatants at 96 hpi and used for NGS. Illumina reads were aligned against the Georgia 2007/1 reference sequence and the estimated average coverage was calculated. Viral DNA from this fraction contained almost no cellular DNA and the percentage of reads aligned against the reference genome increased to 84.89%, with an estimated average coverage of 338× (Table 1).

Interestingly, under these conditions, the analysis from the ASFV Arm/07 virus stock revealed a high number (up to 2216) of sequence variations (Table 2).

**Table 2:** Variant analysis of DNA samples of ASFV Arm/07 from intra- or extracellular virions compared to the Georgia 2007/1 reference genome.

Sample	Total Variants	SNPs	Insertion	Deletions
Arm/07 (DNA from intracellular virions)	6	1	1	4
Arm/07 (DNA from extracellular virions)	2216	2108	58	50

**Figure 1:**
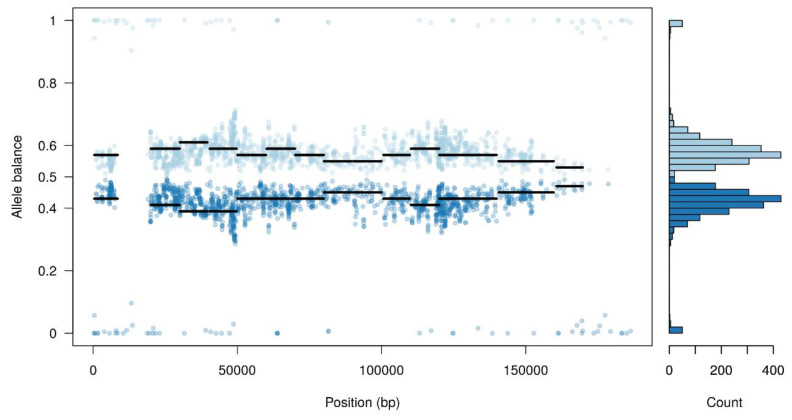
Allele balance distribution of variants from the Arm/07 stock along the Georgia 2007/1 reference genome.

Furthermore, the majority of single nucleotide polymorphisms (SNPs) detected had a high heterozygosity level, where half of the reads supported the reference allele and the other half support an alternate allele (Figure 1). This could suggest the co-existence of at least two different viral populations within the Arm/07 stock.

In view of these results, we asked whether a pool of subpopulations could exist in the Arm/07 wild-type sample stock. A variant calling analysis was performed and a predictive tool was used to separate reads supporting each possible haplotype (as indicated in the Materials and Methods). As expected, one of the presumed subpopulations was very similar to the reference genome, while the other accumulated most of the variants, not resembling the other isolates.

These results might indicate that the high level of total variants found in the Arm/07 sample may arise from a heterogeneous population and this heterogeneity may contain a viral subpopulation (clone) genetically very similar to Georgia 2007/1.

In order to corroborate the co-existence of different populations in the original Arm/07 stock, four independent clones were isolated by plaque assay (after three rounds of purification in COS-1 cells) and further sequenced. It is important to highlight that three successive passages in COS cells of the ASFV strain NH/P68 [50] did not alter the genome sequence relative to the parent stock grown in PAMs. The same variants (0 SNPs, 8 insertions, 3 deletions) compared to the NH/P68 reference sequence (KM262845.1) were detected in the NH/P68 PAM stock before and after the three passages through COS cells (see Supplementary Table S1).

As seen in the Table 3, the percentage of unique aligned reads corresponding to ASFV ranged from 87 to 98% with a mean coverage above 1000× and as high as 6000× in some samples.

The variant calling analysis of the four clones revealed that three of the sequenced clones had a small number of variants when analyzed against the reference genome, suggesting a high similarity to Georgia 2007/1 (Table 4). There was an overall sequence identity of 99.851% between the three most closely related Arm/07 clones, representing the same 8–9 SNPs and insertions/deletions (indels). Importantly, the Arm/07 clone 4 presented an increased number of total variants compared to Georgia 2007/1: 2588 SNPs and 153 indels (Table 4). The high number of nucleotide variations of this clone 4 may greatly contribute to the high number of variations found in the Arm/07 stock (Table 2). Our approach enabled us to identify an additional population very similar to Georgia 2007/1 within the Arm/07 stock, named Arm/07/CBM/c2 clone and another with a high number of variations called Arm/07/CBM/c4.

### 3.2. Arm/07/CBM/c2 Genome Assembly and Variant Calling Analysis of Clones 1 and 3

Next, we focused on characterization of the Arm/07 clones found to have a low number of total nucleotide variations compared to the reference Georgia 2007/1, in particular Arm/07 clone 2 (Arm/07/CBM/c2). This well-established clone will represent our model for successive molecular and functional studies and LAV development.

*De-novo* assembly of the Arm/07/CBM/c2 clone was performed from the Illumina reads, as described in Materials and Methods. The assembled contigs were overlapped to obtain a final assembly of 190,145 bp with a GC content of 38.2%. The presence of a contig corresponding to ITR regions was verified by Sanger sequencing (as explained in Materials and Methods).

**Figure 2:**
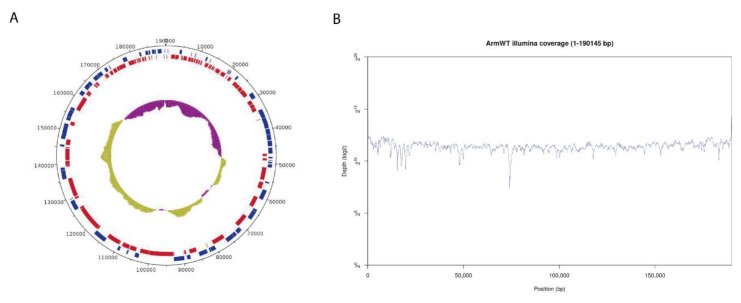
Genome assembly of Arm/07/CBM/c2. (**A**) Circular representation of the genome indicating predicted CDS in “forward” (blue) or “reverse” (red) orientation in the outer circle and the GC/AT content (purple indicates higher AT content, green indicates higher GC content) in the inner circle. (**B**) Whole genome coverage plot using Illumina sequencing reads mapped to the ASFV Arm/07/CBM/c2 assembly.

Genome annotation using PROKKA and GATU software identified 192 CDS features, displayed in a circular representation of the genome (Figure 2A). Genes were labelled and assigned a putative function by homology with the annotated Georgia 2007/1 reference genome. Illumina sequencing reads evenly covered the whole assembled genome with a very homogeneous mean coverage (Figure 2B). Only a few particularly AT-rich regions or G/C homopolymers presented lower coverage but still with enough to retain confidence in the results.

The variant calling analysis of the three similar clones against the assembled genome of Arm/07/CBM/c2 shows very few differences (Table 5); these genetic variants are located in regions with low coverage and difficult resolution.

Given the sequencing depth, the presence of minor variants relative to the Arm/07/CBM/c2 genome was also analyzed. Minor variants are described as genetic variants with an allelic frequency less than 50% and greater than 2%. As shown in Supplementary Figure S1, minor variants of clones 2 and 3 were relatively infrequent and concentrated at both genome ends, matching the repetitive regions with greater variability. Nevertheless, minor variants identified in clone 1 were greater in number and not only concentrated at repetitive regions but throughout the entire genome. It is important to note that the identified minor variants in clone 1 matched with the variants that we found in the clone 4 when comparing the reads of both clones with the reference Georgia 2007/1 genome.

### 3.3. Arm/07/CBM/c4 Genome Assembly Revealed an Unexpected Heterogeneity at the Genome Ends

**Figure 3:**
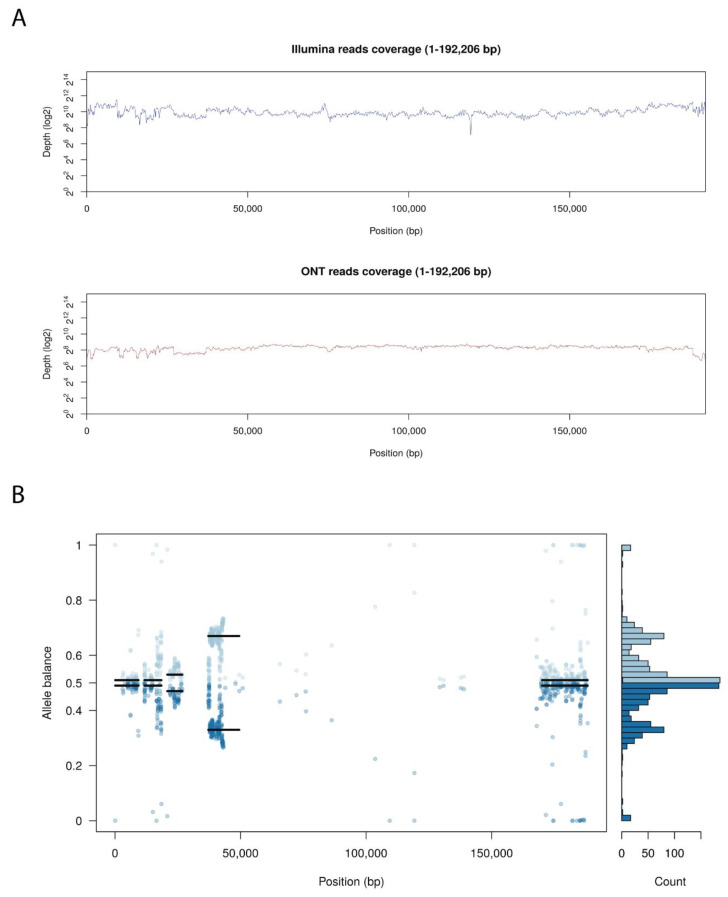
Genome assembly of Arm/07/CBM/c4. (**A**) Whole genome coverage plot using Illumina and Nanopore (ONT) sequencing reads mapped to the ASFV Arm/07 isolate clone 4 assembly. (**B**) Allele balance distribution of variants from Illumina reads along the Arm/07/CBM/c4 assembly.

Arm/07/CBM isolate clone 4 presented an unexpected heterogeneity mostly concentrated at both ends of the genome, analyzing the Illumina reads distribution compare to Georgia 2007/1 genome. Probably due to this reason, we were unable to assemble the whole genome using only the Illumina reads information. Hence, Arm/07/CBM isolate clone 4 was *de-novo* assembled combining both Oxford Nanopore Technology (ONT) and Illumina reads, as described in Material and Methods. ONT reads were polished and corrected with Illumina reads to obtain an assembly of 192,206 bp with a GC content of 38.43%. As can be observed in Figure 3A, both Illumina and ONT reads covered the whole assembled genome. Despite having a lower read depth, long ONT reads show more consistent coverage of the assembly.

Genome annotation of the assembly resulted in 172 CDS features. Genes were labelled and assigned a putative function by identity with different ASFV strains deposited in the databases using again PROKKA and GATU software.

As previously indicated, we observed high levels of heterozygosity mostly concentrated at both ends of Arm/07/CBM/c4 genome, as seen in the plot illustrating the allele balance distribution (Figure 3B). Regions with the highest levels of heterozygosity ranged from 1–26, 37–43 and 168–188 kb. There are also other areas with scattered distributed variants from 58–93 kb and around 140 kb. Nevertheless, most central parts of the genome exhibited low levels of heterozygosity.

### 3.4. Arm/07/CBM/c2 Is Phylogenetically Very Close to Georgia 2007/1 and Other Genotype II Eurasian Strains, While Arm/07/CBM/c4 is Related to Genotype I Strains

In order to characterize the isolated clones Arm/07/CBM/c2 and Arm/07/CBM/c4, we built a phylogenetic tree aligning the CCR of our clones and different ASFV genotype genomes. The CCR region is described as the main conserved region on the ASFV genomes, located between the 5′ end of gene A224L and 3′ end of I196L [20]. Its use in phylogenetic reconstruction allows a higher accuracy of genotype determination, reducing the variability produced by the deletion of several genes in the right and left variable regions on the genome of several attenuated ASFV strains. In addition, in view of the heterogeneity observed in the lateral regions of the Arm/07/CBM/c4 assembled genome, we reasoned that a phylogenetic tree based on the CCR rather than on whole genome sequences would give more valuable information.

**Figure 4:**
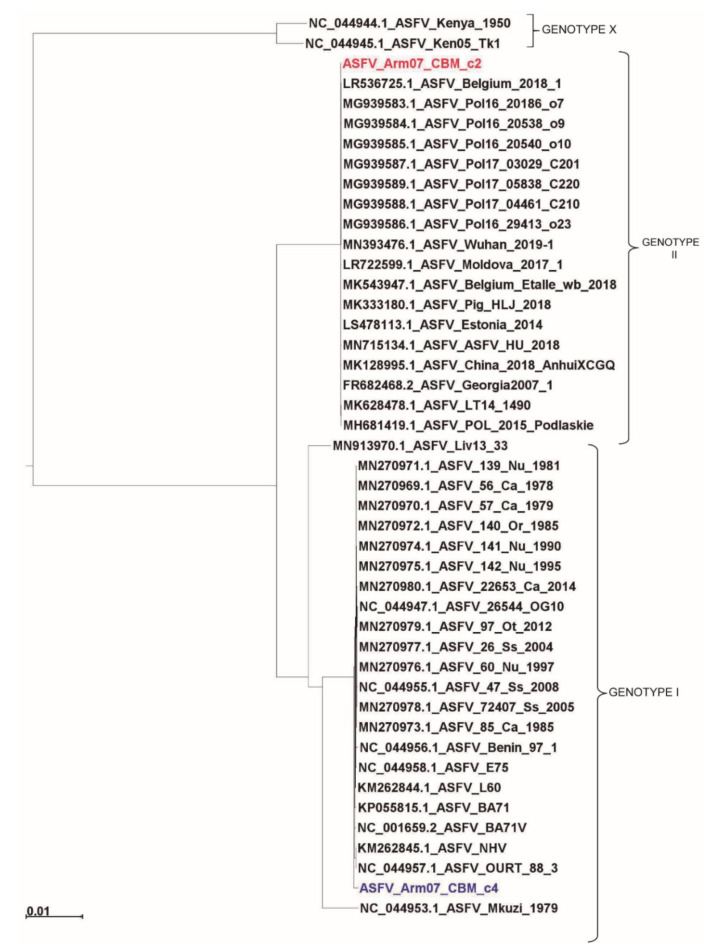
Phylogenetic tree of different ASFV genotypes built by maximum-likelihood method using sequences corresponding to the central conserved region (CCR, comprising 129 kb between A224L and I196L genes). Arm/07/CBM/c2 (highlighted in red) is grouped within the genotype II strains while Arm/07/CBM/c4 (highlighted in blue) is grouped within the genotype I strains. The tree was built based on MAFFT aligned genomes using IqTree software. Automatic settings were used, resulting in the selection of HKY+FK3PU+F+R2 as the best fit model; statistical support of the shown branches was obtained with an ultrafast bootstrap repetition value of 10,000. Substitution per base rate is indicated in the scale below.

As can be observed in Figure 4, our Arm/07/CBM/c2 strain is classified as a genotype II strain, closely related to the reference strain Georgia 2007/1 (LR743116). Remarkably, the isolate Arm/07/CBM/c4 is located in a cluster corresponding to genotype I ASFV genomes but is placed outside the main cluster which contains most of the described genotype I strains, except Mzuki_1979 or Liv13_33. Similar results were obtained when phylogenetic trees were generated based on smaller regions used for phylogenic analysis such as ORFs B602L, B646L, CP204L or EP402R (Supplementary Figure S2).

**Figure 5:**
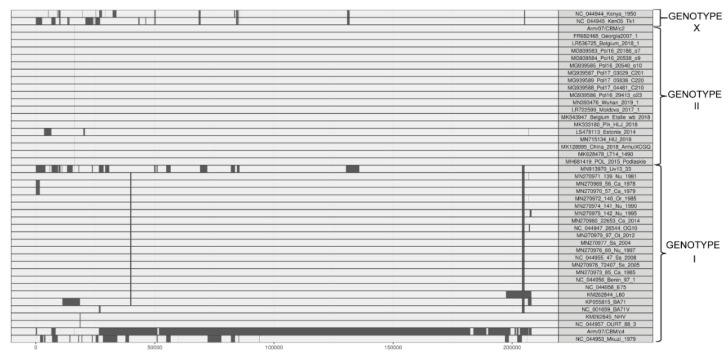
Recombination events inferred by RDP (Recombination Detection Program) in the ASFV genomes used for the phylogenetic tree construction.

A recombination analysis with strains employed for phylogenetic tree was performed similarly to what it is described in Reference [51]. This analysis revealed 99 unique recombination events. Among these, in the Arm/07/CBM/c2 sequence a single recombinant event was found, whereas in Arm/07/CBM/c4 12 recombinant events were found, being Arm/07/CBM/c4 the virus that experienced the higher recombination events followed by ASFV Mkuzi_1979 isolate (Figure 5 and Supplementary Table S2). Furthermore, the two largest recombination regions identified in Arm/07/CBM/c4, ranged from positions 26447-51059 and 51642-179562, covering most of the central region of the genome. In the first of these two regions, the potential parental strains were mainly BA71V, followed by Estonia_2014, while in the second region, the potential both major and minor parental were, respectively, 19 genotype II strains, including Arm/07/CBM/c2 and 18 genotype I strains (Supplementary Table S2). On the other hand, most of the genotype II strains analyzed shared the single recombination event detected in Arm/07/CBM/c2. All together the in silico analysis suggests that Arm/07/CBM/c4 might have be originated from recombinant events from genotype I and II strains.

### 3.5. Arm/07/CBM/c2 Is an ASFV Strain With High Similarity to Georgia 2007/1

According to the above results, the whole genome alignment of all available ASFV genotype II sequences revealed an overall 99.9% nucleotide sequence identity between Arm/07/CBM/c2 and all Asian, Western and Eastern European whole genome sequences (Table 6).

ASFV Arm/07/CBM/c2 showed the highest nucleotide sequence identity to ASFV Georgia 2007/1 (99.992%) among the different genotype II strains tested. The Arm/07/CBM/c2 ASFV strain clusters with the reference isolate Georgia 2007/1 and it is closely related to the Lithuania ASFV/LT14/1490 and Poland ASFV POL/2015/Podlaskie (Figure 4).

Six available whole-genome sequences from genotype II ASFVs were selected for pairwise comparison with the Arm/07/CBM/c2 (marked in bold in Table 6). Arm/07/CBM/c2 showed the highest identity with Georgia 2007/1 (99.992%). Despite the high similarity between both sequences, the start point of the alignment with Georgia 2007/1 was shifted, indicating that the Arm/07/CBM/c2 genome is 218 bp shorter at the 5′ terminal ITR. In addition, Arm/07/CBM/c2 presents a total of nine variations: two SNPs and seven indels. Five of these variants are located in non-coding regions, while two indels and two SNPs are located in the ORFs MGF110-10L-MGF110-14L fusion, ASFV G ACD 00350, NP1450L and E199L (Table 7). The deletion of C-C in Arm/07/CBM/c2 in MGF110-10L-MGF110-14L fusion protein would produce a frameshift mutation that causes the appearance of two different ORFs in Arm/07/CBM/c2, MGF110-11-L and MGF110-14L (Supplementary Figure S3A). In ASFV G ACD 00350, the deletion of GGGG in Arm/07/CBM/c2 would produce a difference in the N-terminal amino acid composition of this protein relative to Georgia 2007/1 (Supplementary Figure S3B). Finally, two SNPs would produce a His104Gln in the E199L protein and a silent mutation (Ser1048) in the NP1450L protein.

From the nine differences observed between Arm/07/CBM/c2 and Georgia 2007/1, a unique variant was found exclusively in ASFV Arm/07/CBM/c2: a nucleotide change (G to A) at position 131,048 resulting in a silent mutation (Ser1048) in the NP1450L protein (Supplementary Table S3). The CC deletion found in the ASFV Arm/07/CBM/c2, which leads to the two separate ORFs 10L and 14L within the MGF110, is present in the Lithuania ASFV/LT14/1490, although the latter has an additional Gly. The deletion of GGGG resulting in a shorter ACD 00190 gene in Arm/07/CBM/c2 is shared with the Lithuania, Poland and Chinese ASFVs (ASFV/LT14/1490, ASFV/POL/2015/Podlaskie, China/2018/AnhuiXCGQ, Wuhan 2019/1). Finally, the Gly127Arg mutation within the E199L found in Arm/07/CBM/c2 is present also in the Chinese and Belgium strains (China/2018/AnhuiXCGQ and Wuhan 2019/1) but absent in ASFV/LT14/1490 and ASFV/POL/2015/Podlaskie. In addition, we identified one poly-C and two poly-G variable sequences located in non-coding regions around positions 1169, 21,582 and 17,628 nucleotides, respectively.

### 3.6. Arm/07/CBM/c4 Showed Significant Differences with Arm/07/CMB/c2 and Presented Sequence Identity Similarity to ASFV Genotype I Strains

Arm/07/CBM/c4 and Arm/07/CBM/c2 yielded less than 98% of identity between them, with 2800 variants distributed along the entire genome (see Supplementary Table S4). This data agrees with the phylogenetic analysis which placed the Arm/07/CBM clones 2 and 4 in different genotypes (II and I respectively). Therefore, the Arm/07/CBM/c4 was compared with the 23 ASFV genotype I genome sequences available in Genbank. The basic alignment of sequenced Arm/07/CBM/c4 against genotype I ASFV strains showed percentages of identities ranging from 96.5–99.6% showing the highest homology to the VERO-cell adapted ASFV Ba71V and the lowest with the South African tick strain Mzuki 1979. The number of variants ranged from 1000–1700 but decreased substantially if only the fixed variants (supported by 100% of the reads) were considered (Table 8).

To gain more information about the phylogenetic relatedness of the sequenced Arm/07/CBM/c4, 10 whole-genome sequences of genotype I ASFVs (marked in bold in Table 8) were used for further pairwise comparison, considering variants supported exclusively by a 100% of frequency.

23 unique variants were found in ASFV Arm/07/CBM/c4. These variants were located within intergenic regions (one single nucleotide mutation and 14 nucleotide indels) and in the ORFs MGF_505-3R (two indels), CP123L (four single nucleotide mutations) and MGF_100-3L (a single nucleotide insertion) (Table 9).

Excluding the unique variants not supported by 100% frequency and those exclusively present in the Arm/07/CBM/c4 sequence, the nucleotide sequence of Arm/07/CBM/c4 showed the highest identity with the virulent ASFV isolate Ba71 with 144 variants, followed by the VERO-cell adapted strain Ba71V (157 variants) and the attenuated Portuguese strains OURT 88/3 and NH/P68 with 159 and 165 variants, respectively. In contrast, the African ASFV strains Mzuki 1979 and Liv13/33 showed the lowest identity when compared with the Arm/07/CBM/c4 (Table 8). Therefore, we focused on nucleotide differences between Arm/07/CBM/c4- and Ba71-encoded proteins and compared them to the remaining strains presented in Table 8, except the South African strains. Compared with the Ba71 virus, the Arm/07/CBM/c4 virus has mutations located in 15 different ORFs. These mutations involve the MGF 110, 360 and 505 members—MGF 110-1L, MGF-6R and 7R, MGF 360-16R and 17R—and the ORFs EP402R, CP123L, I243L and I215L. Among them, 7 were also detected in all the genotype I isolates but none appeared in the Arm/07/CBM/c4 virus (Table 10).

**Figure 6:**
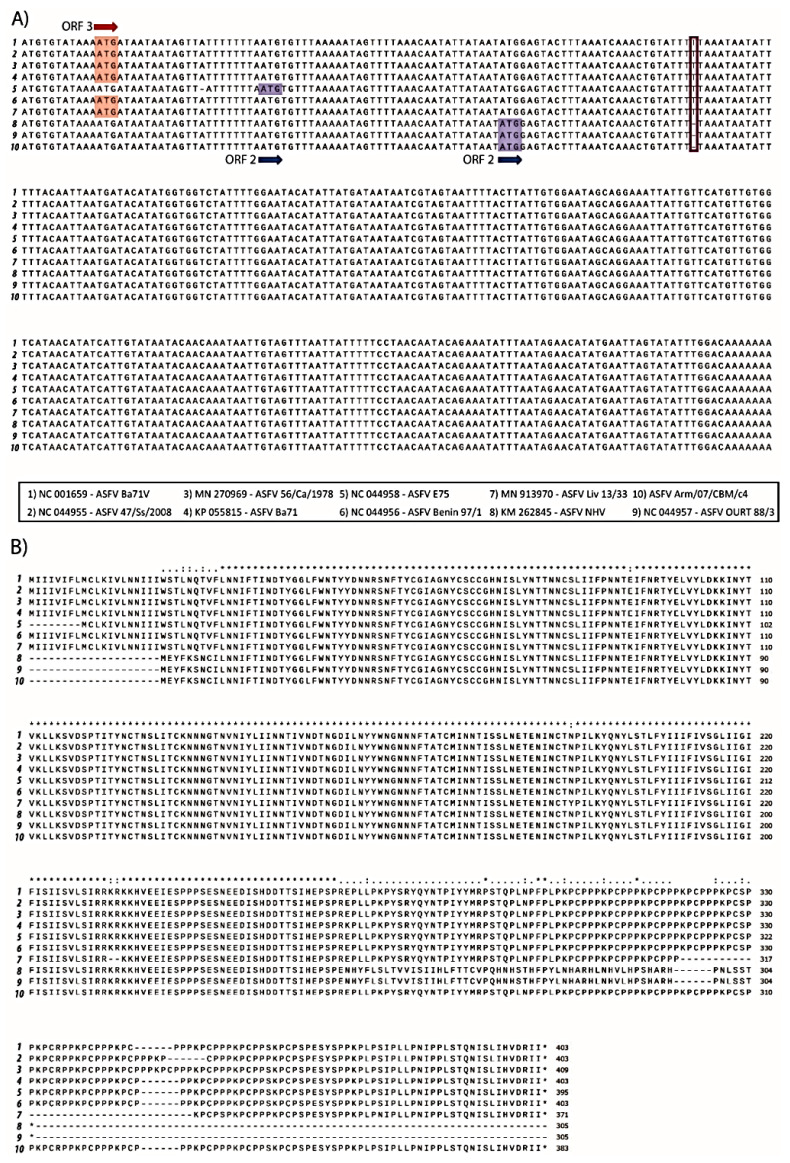
Multiple nucleotide (**A**) and amino acid alignment (**B**) of EP402R sequences corresponding to the genotype I strains numbered 1–9 and Armenia/07/CBM/c4 strain performed using ClustalW. A: Nucleotide alignment of EP402R sequences show that ORF in strains 1–4 and 6 are codified in the third reading frame (ATG in red) whereas ORF for strain 5 and 8–10 is codified in second reading frame (ATG in blue). T deletion identified in Arm/07/CBM/c4 is highlighted by a red square. B: Amino acid alignment of EP402R sequences corresponding to the indicated strains.

Interestingly, we identified a single T deletion at position 75,213, of the EP402R gene in Arm/07/CBM/c4; this nucleotide was present in all the above compared genotype I strains except NH/P68 and OURT 88/3 which contain the deletion (Table 10 and Figure 6A). This mutation was further verified by Sanger sequencing. This deletion provoked a change in the reading frame of EP402R in the Arm/07/CBM/c4, NH/P68 and OURT 88/3, compared to the rest of genotype I strains analyzed, resulting in a shorter N-terminal domain.

EP402R codes for CD2v protein, which is involved in hemoadsorption (HAD) [52] and is one of the hallmarks for virulence. The fact that EP402R in Arm/07/CBM/c4 shared this variant with NH/P68 and OURT 88/3, which are both attenuated and non-hemoadsorbing strains, led us to compare the whole EP402R sequence of Arm/07/CBM/c4, with the corresponding gene sequence among the above strains (Table 10 and Figure 6B). It is noteworthy that the CD2v amino acid sequence of Arm/07/CBM/c4 had a high similarity with the compared genotype I virulent strains, except for the shortened N-terminus, which is identical to that in NH/P68 and OURT 88/3, thus making the CD2v sequence of Arm/07/CBM/c4 unique. These results support the hypothesis that Arm/07/CBM/c4 should be a new emerging ASFV strain.

### 3.7. Arm/07 Clone 2 and Clone 4 Presented Similar Hemoadsorption and Growth Capacity In Vitro

**Figure 7:**
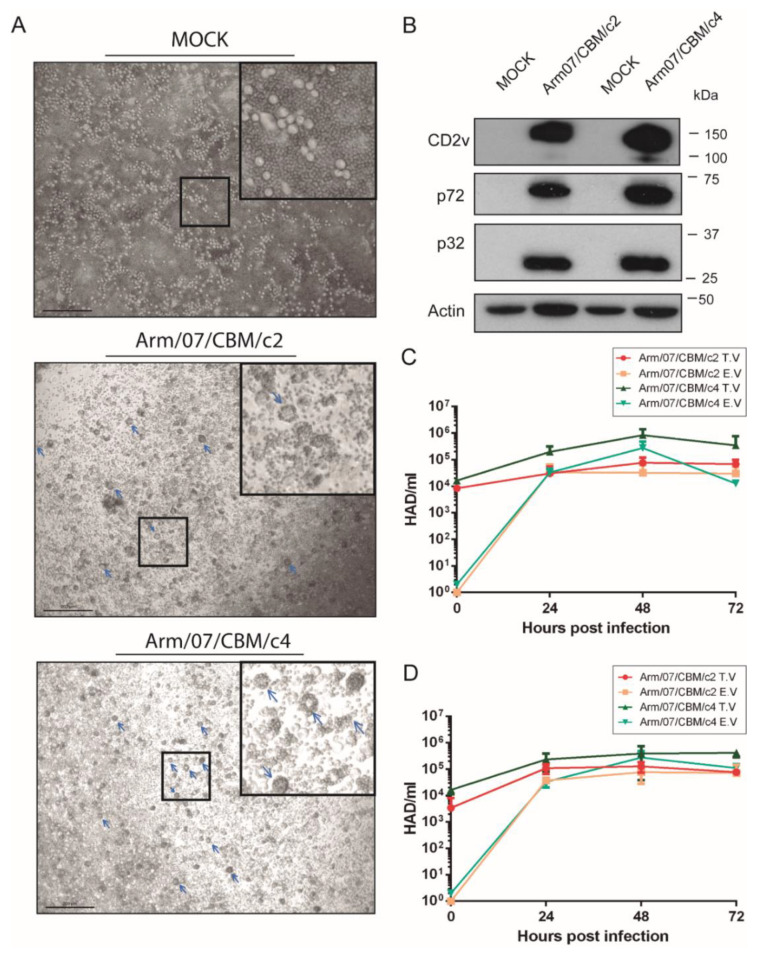
Arm/07/CBM/c2 and Arm/07/CBM/c4 clones express functional CD2v and have a similar capacity for growth in PAMs. PAMs were mock-infected or infected with either Arm/07/CBM/c2 or Arm/07/CBM/c4 (MOI = 0.5) for 16 h. Then, (**A**) porcine erythrocytes were added and after 24 h, rosettes (blue arrows) were detected on a light microscope. (**B**) cells were lysed in RIPA buffer and lysates were separated by 7–20% SDS-PAGE, followed by immunoblotting with anti-CD2v, anti-p72, anti-p32 ASFV proteins or anti-actin. PAMs (**C**) or COS cells (**D**) were infected with either Arm/07/CBM/c2 or Arm/07/CBM/c4 for 24, 48 or 72 h and the total (TV) and extracellular (EV) virus was then assayed for HAD activity. Scale bar in A represents 200 µm.

The absence of functional CD2v is often related to naturally attenuated strains, such as NH/P68 or OURT 88/3 and deletion of the EP402R gene has also been employed to generate LAV prototypes [27,53]. Since we identified in Arm/07/CBM/c4 an EP402R sequence that differed from virulent and attenuated genotype I strains and from genotype II strains (Figure 6), we wondered whether this new EP402R variant would encode a functional CD2v protein. Since the main function of CD2v is related to HAD, we tested Arm/07/CBM/c4’s ability to form rosettes in infected PAMs relative to Arm/07/CBM/c2, which was expected to be HAD(+) by its high similarity with Georgia 2007/1. PAMs were mock-infected or infected with either Arm/07/CBM/c2 or Arm/07/CBM/c4 for 16 h and then porcine erythrocytes were added to the cell culture (as indicated in the Materials and Methods). 24 h later, rosettes were observable by light microscope in PAMs infected with either clone (Figure 7A). Accordingly, CD2v was detected by WB in PAMs infected with either isolate at 16 hpi, in addition to other ASFV proteins such as p72 or p32 (Figure 7B). These results indicate that EP402R codes for a functional CD2v protein in both Arm/07/CBM/c2 and Arm/07/CBM/c4. However, we detected a slightly lower band corresponding to Arm/07/CBM/c4-encoded CD2v, comparing to Arm/07/CBM/c2-encoded CD2v, which may indicate a different patter of post-translational modifications.

To further characterize these clones *in vitro*, we evaluated their ability to grow in PAMs (Figure 7C) and in COS cells (Figure 7D) by analyzing the total (TV) and extracellular virus (EV) produced at 24, 48 and 72 hpi. As shown in Figure 7C, production of Arm/07/CBM/c4 in PAMs was slightly higher than Arm/07/CBM/c2 and no significant differences in the proportion of total vs. extracellular virus was observed between both clones. Similar results were obtained for viruses grown in COS cells (Figure 7D). Hence, there was no appreciable difference between the two isolates in vitro.

### 3.8. Arm/07/CBM/c2 but Not Arm/07/CBM/c4, Prevented IRF3 Phosphorylation and IFN-β Production in PAM

**Figure 8:**
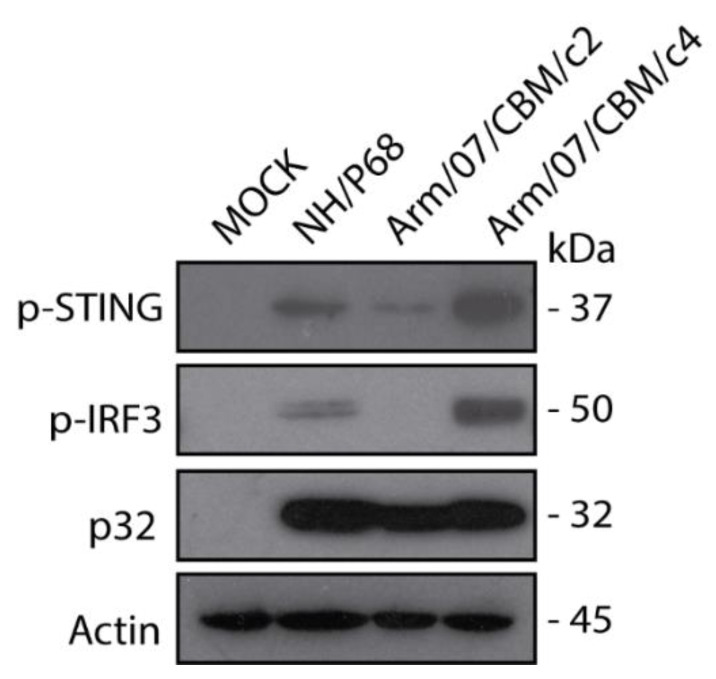
Arm/07/CBM/c2 but not Arm/07/CBM/c4, prevented STING and IRF3 phosphorylation in PAM. PAMs were mock-infected or infected with either Arm/07/CBM/c2, Arm/07/CBM/c4 or NH/P68 strains for 16 h using MOI = 0.5. Cells were lysed in RIPA buffer and lysates were separated by 7–20% SDS-PAGE, followed by immunoblotting with anti-pSTING, anti-p-IRF3, anti-p32 and anti-Actin.

As it has been shown above, Arm/07/CBM clones 2 and 4 presented similar in vitro features, such as grow kinetics and HAD ability. However we were curious about the behavior of these clones in terms of degree of virulence. One of the main differences between ASFV attenuated and virulent strains relies on the ability of ASFV virulent strains to counteract innate immune response through cGAS-STING pathway, whereas ASFV attenuated strain NH/P68 does not control this pathway [54]. Hence, we assessed the ability of Arm/07/CBM/c2 vs. Arm/07/CBM/c4 to counteract the cGAS-STING pathway in vitro. For that, the level of STING and IRF3 phosphorylation in mock-infected and, either Arm/07/CBM/c2 or Arm/07/CBM/c4 infected PAMs, was assessed at 16hpi. NH/P68 was used as attenuated control. As shown in Figure 8 and as expected, NH/P68-infected PAM presented detectable levels of pSTING and p-IRF3. However, both STING and IRF3 phosphorylation were reduced in Arm/07/CBM/c2-infected PAM, indicating that Arm/07/CBM/c2 but no Arm/07/CBM/c4, was able to counteract the cGAS-STING pathway in vitro and suggesting that these strains might display different virulence patterns in vivo.

## 4. Discussion

One of the most realistic short-term strategies for ASFV vaccine development is implementation of LAVs based on the deletion of specific genes from virulent strains [22,23,24]. Thus, exhaustive genetic characterization of both prototypes and parental strains is essential for LAV generation and whole genome sequencing carries obvious advantages over sequencing of individual genes [28].

Interest and technological advances in ASFV genome sequencing have grown over the past few years, allowing for cheaper and more accurate results. Low sequence coverage in older technologies can mask variants or even viral subpopulations in stocks collected from ASFV-infected animals. As shown in Table 1, the proportion of viral DNA in a given extract is a key factor affecting mean coverage in NGS studies. It has been previously reported that low coverage resulted from high levels of eukaryotic DNA contamination [7,9,14,55], which increases the threshold of detection of real nucleotide differences. To avoid this problem, specific PCR-Sanger sequencing (SPSS) has been used to resolve ambiguous or poorly covered areas in the genome, which is time-consuming and may overlook non-homologous subpopulations. As an alternative, several attempts to improve viral DNA purification have been employed for ASFV-NGS, including strain-specific experimentally infected pigs, followed by isolation and sequencing of the virus from the blood of the infected animals [5,8,15]. This method presents obvious economic and animal welfare concerns. There are some other techniques for enrichment of viral vs. cellular DNA, either by nonspecific amplification of DNA [16] or by removal of methylated DNA [56]. Viral DNA capture using specific probes based on known genotype-specific ASFV sequences has also been used as a method to obtain pure viral DNA [12], which in the case of mixed viral populations would still select only the DNA sequences displaying the specific DNA sequences bound by the probes, thus missing any other information present in the sample.

In order to improve the accuracy of the methods to guarantee the nature of the viral stocks that we will use to develop recombinant vaccine prototypes, we present here that use of extracellular virions as the source for viral DNA purification led to a very high viral/eukaryotic DNA ratio (>85%). We obtained greater depth in sequence coverage, sufficient to identify different viral populations within a single stock. In addition, our methodology was able to identify minor variants, defined as variants with an allelic frequency between 2 and 50%, within a single clone (Arm/07 clone 1, Supplementary Figure S1). Minor variants may identify minor sub-populations that could play a role in clinical outcomes of LAV prototypes generated from cell passage. Although other studies have also shown the use of cell-free virus as a genomic source, high percentages of viral DNA and coverage depth necessary for robust data were not finally obtained [7,9]. It is uncertainly whether the differences lie in the NGS technology used (Roche 454 GS FLX) [9] or possibly to other difficulties in the accomplished methodology [7].

In our hands, the Arm/07 stock, which was thought to be composed of a homogeneous viral population, was unexpectedly found to include viral genomic heterogeneity. In order to characterize the viral populations detected, we further pursued isolation of individual clones using plaque purification in COS-1 cells. It is largely known that growth of ASFV strains in cells other than their natural targets, that is, PBMs/PAMs, can induce genomic modifications. For instance, sequencing of the whole genome of Ba71-adapted in Vero cells revealed a multitude of changes compared to its parental strain [6,57]. Other ASFV strains have also undergone genetic mutation when grown in certain cell lines, such as Vero or CV1 [58,59]. Importantly, we have verified that isolation of Arm/07/CBM/c2 by three passages in COS-1 did not generate variations in its sequence. This finding is further supported by the fact that a low number of passages (typically three) in COS-1 cells did not produce any remarkable alteration in the genome of ASFV NH/P68 (Supplementary Table S1). In addition, it has previously been reported that infection of COS-1 cells did not induce genome modifications after 20 passages of a Ba71 virulent-derived LAV [27].

This study describes for the first time the sequence of the ASFV Arm/07 genome, using a workflow with an emphasis on non-biased viral DNA purification. Starting from cell-free infection supernatants enabled us to obtain high quality sequences and coverage depth while minimizing cellular DNA contamination, revealing an unexpected heterogeneity in an ASFV Arm/07 stock which might have been missed using standard NGS workflows [12]. Plaque isolation confirmed the existence of at least two distinct viral populations within the original Arm/07 stock. The origin of these viral populations is currently unknown. It is plausible that these populations co-infected the same animal. Indeed, co-infections by two different isolates belonging to the same genotype, in a single animal have been previously found [60].

The first sub-population was represented by the clone named Arm/07/CBM/c2 and has been fully characterized. The sequence identity that this clone Arm/07/CBM/c2 shares with the lastly updated sequence Georgia 2007/1 [12], the currently circulating member of genotype II, is not surprising, as both are geographically close isolates and ASFV is a large DNA virus whose mutation rate is expected to be low. The second isolated clone, named Arm/07/CBM/c4, has been also characterized, showing a CCR homology compatible with ASFV genotype I. The Arm/07/CBM/c4 presented a high heterogeneity at both ends of the genome but only the variants that were fixed in the assembled genome (supported by ~100% of the reads) were taken into account for pairwise comparison with other genotype I strains.

From these fixed variants, Arm/07/CBM/c4 presented 23 unique variants compared with all genotype I strains displayed in Table 8, thus making Arm/07/CBM/c4 a distinctive strain. This fact may discard the possibility of a laboratory contamination with any of the known genotype-I strains commonly used in the ASFV labs. It is further noteworthy that Arm/07/CBM/c4 compared to some of the most widely strains used in the ASFV field labs, such as Ba71V, OURT 88/3 and NH/P68, the number of 179, 181 and 187 fixed variants were respectively found (see Table 8). Moreover, the in silico analysis revealed that recombination events, in vivo and/or in vitro, might have a role in the origin of Arm/07/CBM/c4. Even more interestingly, a single mutation found at the N-terminal region of the EP402R gene (CD2v) of Arm/07/CBM/c4, further assessed by NGS and Sanger, induced a frameshift variant that shortened the N-terminal domain of CD2v. The overall sequence of CD2 of Arm/07/CBM/c4 resulted to be unique, which is relevant since we also demonstrated that this virus is HAD+. Hemadsorption and virulence are two concepts that have been traditionally linked in ASFV [61,62]. Studies concerning the CD2v sequence and structure of Arm/07/CBM/c4 linked to HAD might be of great interest for the ASFV community. Not only that but also, our results showed that both clones have different ability to modulate innate immune response in vitro. While Arm/07/CBM/c2 is able to counteract STING and IRF3 phosphorylation, in Arm/07/CBM/c4-infected cells we detected pSTING and pIRF3 in a similar manner that the attenuated NH/P68. These results suggest that Arm/07/CBM/c4 induced the cGAS-STING pathway and may provoke an attenuated phenotype *in vivo*, indicating that that may be an adequate model for LAV development in the near future, although in vivo experiments should be performed in order to verify this hypothesis. Due to the heterogeneity in the left and right ends of Arm/07/CBM/c4, a region that encodes genes reported to be important in immune evasion [63,64], individual Arm/07/CBM/c4-derived clones will be obtained, which may be useful in future vaccine clinical trials based on their special characteristics and putative alteration in degree of virulence that will be analyzed in pigs in the near future.

## 5. Conclusions

This study describes for the first time the sequence of the ASFV Arm/07 genome, using a workflow with an emphasis on non-biased viral DNA purification. This powerful methodology is key for genetic characterization of ASFV stock and LAV prototypes, as can reveal viral variants previously not expected. Two separate Arm/07/CBM clones 2 and 4, belonging to genotype II and I respectively, have been identified at the Arm/07 stock. Due to their specific characteristics regarding hemadsorption and immune behavior, we speculate that Arm/07/CBM/c2 and Arm/07/CBM/c4 may display different degrees of virulence in animals. Both isolates will be ASFV prototypes for further molecular studies and it is challenging that Arm/07/CBM/c4, could show a more attenuated phenotype and would represent a candidate for future LAV development.

## Figures and Tables

**Table 3:** Illumina sequencing statistics of ASFV clones isolated from Arm/07 stock.

Sample	Total Reads	Aligned Reads	% Aligned Reads	Mean Coverage
Arm/07 isolated clone 1	1,356,319	1,261,599	93.02	1495
Arm/07 isolated clone 2	2,693,332	2,365,480	87.83	2801
Arm/07 isolated clone 3	5,443,265	5,365,134	98.56	6094
Arm/07 isolated clone 4	968,957	906,978	93.6	1049

**Table 4:** Variant analysis of DNA samples of ASFV clones isolated from the Arm/07 stock compared to Georgia 2007/1 genome.

Sample	Total Variants	SNPs	Insertions	Deletions
Arm/07 isolated clone 1	9	2	2	5
Arm/07 isolated clone 2	8	2	2	4
Arm/07 isolated clone 3	9	2	3	4
Arm/07 isolated clone 4	2741	2588	81	72

**Table 5:** Variant analysis of DNA samples of ASFV clones isolated from the Arm/07 stock compared to Arm/07/CBM/c2 genome.

Sample	Total Variants	SNPs	Insertions	Deletions
Arm/07 isolated clone 1	2	0	1	1
Arm/07 isolated clone 2	1	0	1	0
Arm/07 isolated clone 3	2	0	2	0

**Table 6:** Percentage of identity and number of variants found in Arm/07/CBM/c2 compared to other genotype II strains. Variants found in the strains indicated in bold are described in detail in Supplementary Table S3.

GB Accession Number—ASFV Strain	Percentage of Identity (%)	Number of Variants vs. Arm/07/CBM/c2
**LR743116 ASFV Georgia 2007/1**	**99.992**	**9**
LS478113 Estonia 2014	99.986	9
MG939586 Pol16_29413_o23	99.986	17
MK543947 Belgium/Etalle/wb/2018	99.985	13
**LR536725 Belgium 2018/01**	**99.984**	**13**
LR722599 ASFV Moldova2017/1	99.982	14
**MK128995 China/2018/AnhuiXCGQ**	**99.982**	**17**
MN715134ASFV_HU_2018	99.982	17
**MH681419 ASFV/POL/2015/Podlaskie**	**99.982**	**22**
MK333180 Pig/HLJ/2018	99.979	16
MK333181 DB/LN/2018	99.979	16
MG939588 Pol17_04461_C210	99.978	16
MG939583 Pol16_20186_o7	99.977	22
MG939585 Pol16_20540_o10	99.976	22
**MK628478 ASFV/LT14/1490**	**99.975**	**21**
MN172368 ASFV/pig/China/Cas19-01/2019	99.975	16
MG939587 Pol17_03029_C201	99.975	21
MG939589 Pol17_05838_C220	99.974	18
**MN393476 ASFV Wuhan 2019-1**	**99.970**	**19**
MG939584 Pol16_20538_09	99.958	51

**Table 7:** Mutations observed on the alignment of ASFV Arm/07/CBM/c2 with the reference genome ASFV Georgia 2007/1 and their effect on diverse coding sequences.

Position	Gene	Mutation	Effect
1169	Non-coding region	C Deletion	Indel
14,014	MGF110-10L-MGF110-14L	Deletion 2xC	Alternative ORFs
15,460	Non-coding region	Insertion 2xC	Indel
17,412	Non-coding region	Insertion 2xG	Indel
17,628	Non-coding region	G Insertion	Indel
19,580	Non-coding region	G Deletion	Indel
19,785	ASFV_G_ACD_00350	Deletion 4xG	Change in N-terminal amino acid
131,048	NP1450	G → A	Silent mutation Ser 1048
166,908	E199L	T → G	Gln 104 His

**Table 8:** Percentage identity between ASFV Arm/07/CBM/c4 strain and different genotype I ASFV strains and number of total and fixed variants.

GB Accession Number—ASFV Strain	Percentage Identity (%)	Number of Variants vs. Arm/07/CBM/c4	
		Total Variants	Fixed Variants
**NC 001659 ASFV BA71V**	99.605	1011	179
MN270974 ASFV 141/Nu/1990	99.558	1708	332
MN270973 ASFV 85/Ca/1985	99.556	1691	318
MN270975 ASFV 142/Nu/1995	99.556	1711	335
MN270978 ASFV 72407/Ss/2005	99.553	1705	329
MN270976 ASFV 60/Nu/1997	99.553	1703	327
MN270977 ASFV 26/Ss/2004	99.551	1726	349
**NC 044955 ASFV 47/Ss/2008**	99.549	1731	354
MN270979 ASFV 97/Ot/2012	99.549	1724	346
NC 044947 ASFV 26544/OG10	99.548	1721	346
MN270980 ASFV 22653/Ca/2014	99.547	1724	344
MN270971 ASFV 139/Nu/1981	99.520	1686	314
**MN270969 ASFV 56/Ca/1978**	99.519	1685	313
MN270970 ASFV 57/Ca/1979	99.518	1684	312
KM262844 ASFV L60	99.515	1681	311
MN270972 ASFV 140/Or/1985	99.515	1689	317
**KP055815 ASFV BA71**	99.504	1420	166
**NC 044958 ASFV E75**	99.503	1673	338
**KM262845 ASFV NHV (NH/P68)**	99.464	1366	187
**NC 044957 ASFV OURT 88/3**	99.458	1364	181
**NC 044956 ASFV Benin 97/1**	99.140	1744	363
**MN913970 ASFV Liv13/33**	98.140	3663	2000
**NC 044953 ASFV Mkuzi_1979**	96.542	3581	1799

**Table 9:** Unique mutations observed in the alignment of ASFV Arm/07/CBM/c4 with the selected ASFV strains from Table 8 (in bold).

Position	Gene	Variant	Effect
5008	Intergenic region	CT deletion	
7671	Intergenic region	A deletion	
23,639	Intergenic region	T deletion	
23,643	Intergenic region	T →A	
37,409	MGF 505-3R	T insertion	Frameshift variant: Trp278
37,415	MGF 505-3R	T deletion	Premature STOP codon
37,459	Intergenic region	T deletion	
37,483	Intergenic region	A insertion	
119,241	CP123L	GG → TT	Lys 48 Ser
119,250	CP123L	CC → GA	Leu 45 Pro
170,324	Intergenic region	A deletion	
171,591	Intergenic region	A insertion	
171,594	Intergenic region	A insertion	
174,208	Intergenic region	C deletion	
179,899	Intergenic region	A deletion	
182,231	Intergenic region	A deletion	
182,242	Intergenic region	GTT deletion	
182,246	Intergenic region	C deletion	
182,282	Intergenic region	T deletion	
182,611	MGF 100-3L	G deletion	Frameshift variant: Lys48

**Table 10:** Unique mutations found within open reading frames of Arm/07/CBM/c4 relative to genotype I strains (excluding Mkuzi 1979 and Liv13/33).

Position	Variant	Gene	BA71	BA71V	OURT 88/3	NHV (NH/P68)	E75	L60	Ca 1978	Ss 2008	Benin 97
8177	G/GTTA	MGF_110-1L	Yes	Yes	Yes	Yes	Yes	Yes	Yes	Yes	Yes
41,704	T/A	MGF_505-6R	Yes	No	Yes	Yes	Yes	Yes	Yes	Yes	Yes
42,392	GC/G	MGF_505-6R	Yes	No	Yes	Yes	Yes	Yes	Yes	Yes	Yes
42,395	TA/T	MGF_505-6R	Yes	No	Yes	Yes	Yes	Yes	Yes	Yes	Yes
42,397	TG/T	MGF_505-6R	Yes	No	Yes	Yes	Yes	Yes	Yes	Yes	Yes
42,410	T/TT	MGF_505-6R	Yes	No	Yes	Yes	Yes	Yes	Yes	Yes	Yes
43,210	A/ATAT	MGF 505-7R	Yes	No	Yes	Yes	Yes	Yes	Yes	Yes	Yes
75,213	T/TT	EP402R	Yes	Yes	No	No	Yes	Yes	Yes	Yes	Yes
119,237	TTT/T	CP123L	Yes	Yes	Yes	Yes	Yes	Yes	Yes	Yes	Yes
119,243	T/A	CP123L	Yes	Yes	Yes	Yes	Yes	Yes	Yes	Yes	Yes
119,244	T/G	CP123L	Yes	Yes	Yes	Yes	Yes	Yes	Yes	Yes	Yes
173,579	T/TT	I243L	Yes	Yes	Yes	Yes	Yes	Yes	Yes	Yes	Yes
176,214	T/TC	I215L	Yes	Yes	Yes	Yes	Yes	Yes	Yes	Yes	Yes
179,693	A/AA	MGF_360-16R	Yes	No	No	No	No	No	No	No	No
185,623	T/TCG	MGF 360-17R	Yes	Yes	Yes	Yes	Yes	Yes	Yes	Yes	Yes

## Supplementary Materials

The following are available online at https://www.mdpi.com/2076-393X/8/4/625/s1, Figure S1: Minor variants from Arm/07 clone 1 (A), clone 2 (B) and clone 3 (C) compared to the Arm/07/CBM/c2 assembled genome. Blue dots represent SNPs and red dots represent indels, Figure S2. Phylogenetic trees build by maximum-likelihood method using sequences extracted from Armenia/07/CBM/c2, Armenia/07/CBM/c4 and different genotype I, II and X strains, corresponding to genes: A) B602L, B) B646L (p72 protein), C) CP204L (p32 protein) and D) EP402R (CD2v protein). Substitution per base rate is marked below each tree, Figure S3: Analysis of (A) MGF-110-11L/14L and (B) ASFV_G_ACD_00350 sequences of Armenia/07/CBM/c2 compared to Georgia 2007/1 (FR286428.2) homologous genes. A: Deletion of two cytosines in Arm/07/CBM/c2 leaded to the split of the single Georgia 2007/1 MGF-110-10L-14L fusion protein (indicated in green) in two overlapping ORFs in Armenia/07/CBM/c2: MGF-110-11L (indicated in red) and MGF-110-14L (indicated in blue). Left: diagram (above) and amino acid alignment (below). Right: nucleotide alignment showing the CC deletion (red square). B: Deletion of four guanines in Arm/07/CBM/c2 induced a frameshift generating an N-terminus variation comparing to Georgia 2007/1 ASFV_G_ACD_00350. Left: diagram (above) and amino acid alignment (below). Right: nucleotide alignment showing the GGGG deletion (red square). Alignments were performed by ClustalW and visualized by Snapgene software, Table S1: Variant analysis of an NH/P68 stock grown in PAMs, before and after three passages in COS-1 cells compared to the NH/P68 reference sequence (KM262845.1), Table S2. Recombination events detected by at least two methods in RDP. Recombination region, potential major and minor parents and methods supporting the events are shown. [Excel File], Table S3. List of SNPs and indels obtained from alignment (using Nucmer) of Arm/07/CBM/c2 with Georgia 2007/1, ASFV/LT14/1490, ASFV/POL/2015/Podlaskie, China/2018/AnhuiXCGQ, Belgium 2018/01 and ASFV Wuhan 2019/1, Table S4: List of SNPs and indels obtained from alignment of Arm/07/CBM/c2 with Arm/07/CBM/c4 using Nucmer. [Excel File].
