# Supplementary material for: Identification and Isolation of Two Different Subpopulations Within African Swine Fever Virus Arm/07 Stock
Source: Vaccines (Basel). 2020 Oct 25;8(4):625. doi: 10.3390/vaccines8040625 (PMC7712101; doi:10.3390/vaccines8040625)
Supplement: Supplementary file 1 [file vaccines-08-00625-s001.zip › vaccines-950602 proof sup/vaccines-950602 proof sup .docx]

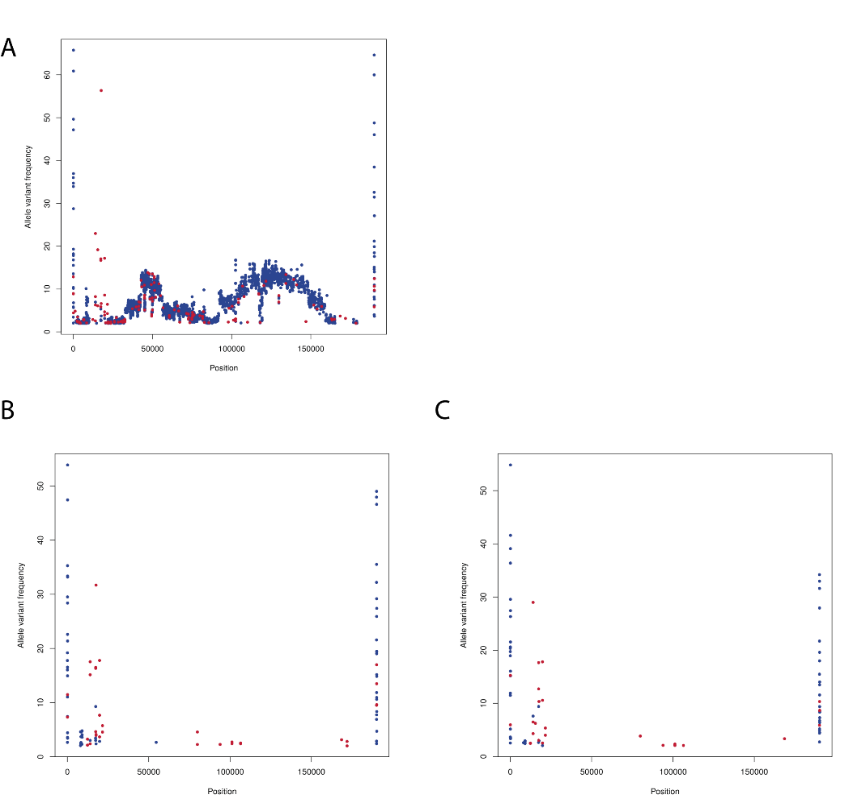


**Supplementary** **Figure S1.** Minor variants from Arm/07 clone 1 (**A**), clone 2 (**B**) and clone 3 (**C**) compared to the Arm/07/CBM/c2 assembled genome. Blue dots represent SNPs and red dots represent indels.


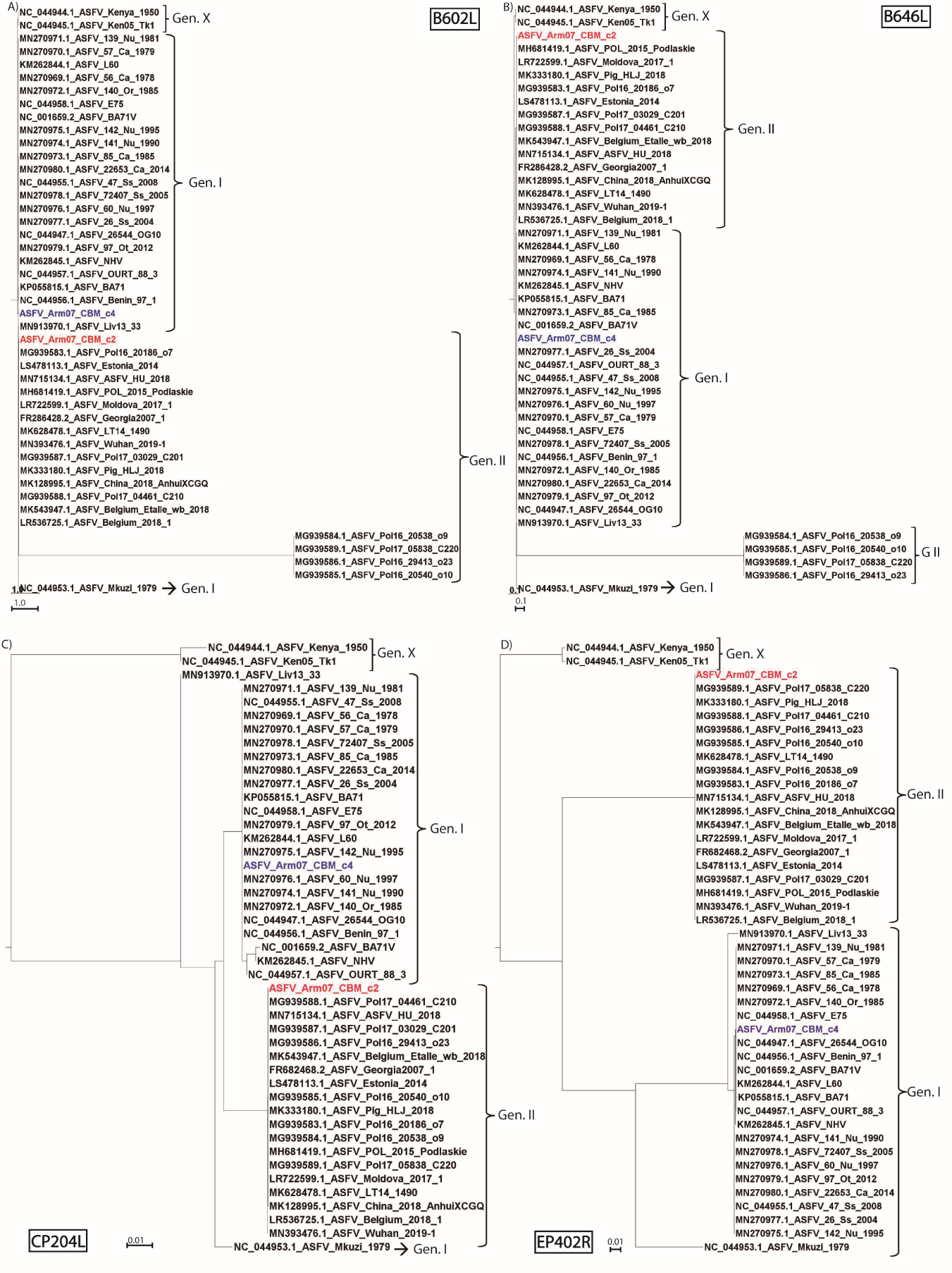


**Supplementary** **Figure S2.** Phylogenetic trees build by maximum-likelihood method using sequences extracted from Armenia/07/CBM/c2, Armenia/07/CBM/c4 and different genotype I, II and X strains, corresponding to genes: **A**) B602L, **B**) B646L (p72 protein), **C**) CP204L (p32 protein) and **D**) EP402R (CD2v protein). Substitution per base rate is marked below each tree.


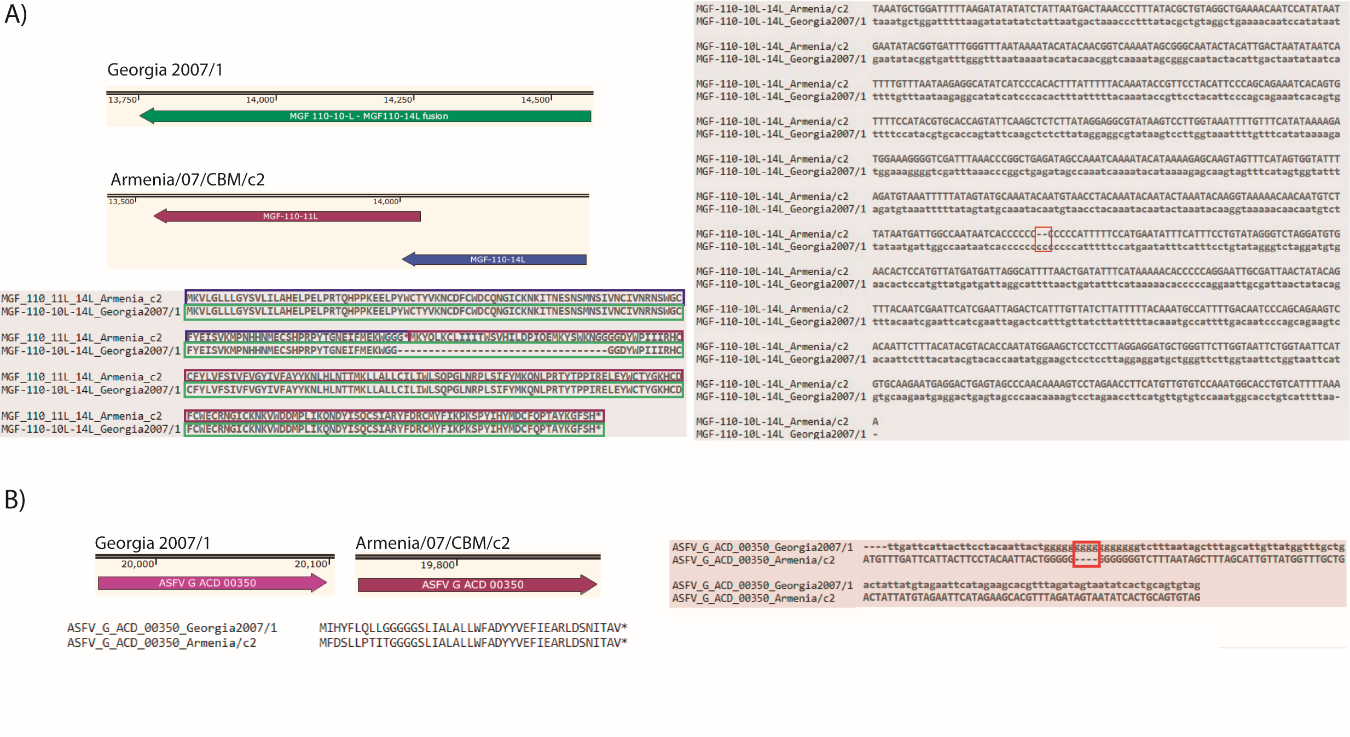


**Supplementary Figure S3.** Analysis of (A) MGF-110-11L/14L and (B) ASFV_G_ACD_00350 sequences of Armenia/07/CBM/c2 compared to Georgia 2007/1 (FR286428.2) homologous genes. A: Deletion of two cytosines in Arm/07/CBM/c2 leaded to the split of the single Georgia 2007/1 MGF-110-10L-14L fusion protein (indicated in green) in two overlapping ORFs in Armenia/07/CBM/c2: MGF-110-11L (indicated in red) and MGF-110-14L (indicated in blue). Left: diagram (above) and amino acid alignment (below). Right: nucleotide alignment showing the CC deletion (red square). B: Deletion of four guanines in Arm/07/CBM/c2 induced a frameshift generating an N-terminus variation comparing to Georgia 2007/1 ASFV_G_ACD_00350. Left: diagram (above) and amino acid alignment (below). Right: nucleotide alignment showing the GGGG deletion (red square). Alignments were performed by ClustalW and visualized by Snapgene software.

**Supplementary Table S1.** Variant analysis of an NH/P68 stock grown in PAMs, before and after three passages in COS-1 cells compared to the NH/P68 reference sequence (KM262845.1).

| **Sample** | **Mean Coverage** | **Total variants** | **SNPs** | **Insertions** | **Deletions** |
| --- | --- | --- | --- | --- | --- |
| NH/P68 (PAM) | 7058 | 11 | 0 | 8 | 3 |
| NH/P68 (3x COS-1) | 878 | 11 | 0 | 8 | 3 |

**Supplementary Table S2.** Recombination events detected by at least two methods in RDP. Recombination region, potential major and minor parents and methods supporting the events are shown. **[**Excel File].

**Supplementary Table S3.** List of SNPs and indels obtained from alignment (using Nucmer) of Arm/07/CBM/c2 with Georgia 2007/1, ASFV/LT14/1490, ASFV/POL/2015/Podlaskie, China/2018/AnhuiXCGQ, Belgium 2018/01 and ASFV Wuhan 2019/1.

| **Position** | **Localization** | **Type** | **Mutation** | **Description** | **Strain(s) Where Variant is Found** |
| --- | --- | --- | --- | --- | --- |
| 1169 | Non-coding region | Indel | Deletion C/CC/CCC/CC/CC |  | LR713116 ASFV Georgia 2007/1, MK628478 ASFV/LT14/1490, MH681419 ASFV/POL/2015/Podlaskie, LR536725 Belgium 2018/01, MN393476 ASFV Wuhan 2019-1 |
| 2742 | Non-coding region | Indel | T Insertion |  | MK128995 China/2018/AnhuiXCGQ, MN393476 ASFV Wuhan 2019-1 |
| 6562 | Non-coding region | Indel | T Insertion |  | MK128995 China/2018/AnhuiXCGQ, LR536725 Belgium 2018/01, MN393476 ASFV Wuhan 2019-1 |
| 6840 | MGF 110-1L CDS | SNP | T- > C | STOP_gained Trp197 * | MK128995 China/2018/AnhuiXCGQ, LR536725 Belgium 2018/01, MN393476 ASFV Wuhan 2019-1 |
| 12357 | ASFV G ACD 00190 | Indel | A Insertion | Frameshift_variant  Ser5 | MK628478 ASFV/LT14/1490, MH681419 ASFV/POL/2015/Podlaskie, MK128995 China/2018/AnhuiXCGQ, MN393476 ASFV Wuhan 2019-1 |
| 14014 | MGF 110-10-L - MGF110-14L | Indel | Deletion CC/CCC/CC  C Insertion | Frameshift_variant  Asp12/  Conservative inframe_insertion  Gly8/  Frameshift_variant  Gly11 | LR713116 ASFV Georgia 2007/1, MK628478 ASFV/LT14/1490, LR536725 Belgium 2018/01, MN393476 ASFV Wuhan 2019-1 |
| 15452 | Non-coding region | Indel | Insertion CCCCCCCCCC |  | MK628478 ASFV/LT14/1490 |
| 15456 | Non-coding region | Indel | Insertion CCCCCC |  | MN393476 ASFV Wuhan 2019-1 |
| 15459 | Non-coding region | Indel | Insertion CCC |  | MH681419 ASFV/POL/2015/Podlaskie, MK128995 China/2018/AnhuiXCGQ |
| 15460 | Non-coding region | Indel | Insertion CC |  | LR713116 ASFV Georgia 2007/1, MK628478 ASFV/LT14/1490, MH681419 ASFV/POL/2015/Podlaskie, MK128995 China/2018/AnhuiXCGQ |
| 15461 | Non-coding region | Indel | C Deletion |  | LR536725 Belgium 2018/01 |
| 17403 | Non-coding region | Indel | Insertion GGGGG |  | MN393476 ASFV Wuhan 2019-1 |
| 17410 | Non-coding region | Indel | Insertion GGGG |  | MK128995 China/2018/AnhuiXCGQ |
| 17412 | Non-coding region | Indel | Insertion GG |  | LR713116 ASFV Georgia 2007/1, MK128995 China/2018/AnhuiXCGQ |
| 17628 | Non-coding region | Indel | Insertion G/  Insertion G/Deletion GG/  Insertion G  Insertion G |  | LR713116 ASFV Georgia 2007/1, MH681419 ASFV/POL/2015/Podlaskie, MK128995 China/2018/AnhuiXCGQ, MN393476 ASFV Wuhan 2019-1 |
| 19574 | Non-coding region | Indel | G Deletion |  | MK628478 ASFV/LT14/1490 |
| 19580 | Non-coding region | Indel | Deletion G/ GG |  | LR713116 ASFV Georgia 2007/1, MH681419 ASFV/POL/2015/Podlaskie |
| 19783 | ASFV G ACD 00350 | Indel | Insertion GGG | Conservative_inframe  insertion: Gly14 | MH681419 ASFV/POL/2015/Podlaskie |
| 19784 | ASFV G ACD 00350 | Indel | Insertion GG | Frameshift_variant  Gly14 | MK628478 ASFV/LT14/1490, MH681419 ASFV/POL/2015/Podlaskie |
| 19785 | ASFV G ACD 00350 | Indel | Deletion GGGG/ G/GG | Frameshift_variant  Ser15 | LR713116 ASFV Georgia 2007/1, MK128995 China/2018/AnhuiXCGQ, LR536725 Belgium 2018/01 |
| 21582 | Non-coding region | Indel | Deletion GGGG/G/G/ GG/G |  | MK628478 ASFV/LT14/1490, MH681419 ASFV/POL/2015/Podlaskie, MK128995 China/2018/AnhuiXCGQ, LR536725 Belgium 2018/01, MN393476 ASFV Wuhan 2019-1 |
| 26204 | MGF 360-10L | SNP | C- > T | Ser329Asn | MK128995 China/2018/AnhuiXCGQ, MN393476 ASFV Wuhan 2019-1 |
| 26788 | MGF 360-10L | SNP | G- > A | Silent mutation  Cys134Cys | MK628478 ASFV/LT14/1490, MH681419 ASFV/POL/2015/Podlaskie |
| 27205 | Non-coding region | Indel | T Insertion |  | LR536725 Belgium 2018/01 |
| 32819 | MGF 360-14L | Indel | C Deletion | Frameshift_variant  Ala284 | MN393476 ASFV Wuhan 2019-1 |
| 39776 | Non-coding region | Indel | Deletion T |  | MH681419 ASFV/POL/2015/Podlaskie |
| 44355 | MGF 505-9R | SNP | G- > A | Glu323Lys | MK128995 China/2018/AnhuiXCGQ, LR536725 Belgium 2018/01, MN393476 ASFV Wuhan 2019-1 |
| 62957 | F1055L | SNP | 1. > T | Val268 Glu | MH681419 ASFV/POL/2015/Podlaskie |
| 69288 | EP1242L | SNP | A- > G | Silent mutation  Ile573Ile | MK628478 ASFV/LT14/1490, MH681419 ASFV/POL/2015/Podlaskie |
| 73048 | Non-coding region | Indel | T Insertion |  | LR536725 Belgium 2018/01 |
| 74030 | EP153R | Indel | Deletion AAAAAC | Conservative_inframe  deletion: His149insLysAsn- > Lys148 | MN393476 ASFV Wuhan 2019-1 |
| 83647 | C717R | SNP | G- > T | Gly266 Val | MN393476 ASFV Wuhan 2019-1 |
| 100401 | B354L | SNP | A- > G | Silent mutation  Ser348Ser | MH681419 ASFV/POL/2015/Podlaskie |
| 103092 | Non-coding region | Indel | G Deletion |  | MK128995 China/2018/AnhuiXCGQ, MN393476 ASFV Wuhan 2019-1 |
| 129197 | O174L | SNP | A- > G | Ser110 Pro | MK128995 China/2018/AnhuiXCGQ |
| 129301 | O174L | SNP | A- > G | Phe75 Ser | MK128995 China/2018/AnhuiXCGQ |
| 129326 | O174L | SNP | A- > G | Ser67 Pro | MK128995 China/2018/AnhuiXCGQ |
| 131048 | NP1450L | SNP | G- > A | Silent mutation  Ser1048Ser | LR713116 ASFV Georgia 2007/1, MK628478 ASFV/LT14/1490, MH681419 ASFV/POL/2015/Podlaskie, MK128995 China/2018/AnhuiXCGQ, LR536725 Belgium 2018/01, MN393476 ASFV Wuhan 2019-1 |
| 134293 | NP419L | SNP | C- > T | Ser414Asn | MK128995 China/2018/AnhuiXCGQ, LR536725 Belgium 2018/01, MN393476 ASFV Wuhan 2019-1 |
| 144849 | D117L | SNP | A- > G | Leu84 Pro | LR536725 Belgium 2018/01 |
| 149547 | P1192R | SNP | A- > G | Ile415 Val | MK628478 ASFV/LT14/1490 |
| 156002 | Non-coding region | SNP | T- > C |  | MK628478 ASFV/LT14/1490, MH681419 ASFV/POL/2015/Podlaskie |
| 156670 | H240R | SNP | A> G | His186 Arg | MK628478 ASFV/LT14/1490, MH681419 ASFV/POL/2015/Podlaskie |
| 166493 | Non-coding region | SNP | T- > C |  | MK628478 ASFV/LT14/1490, MH681419 ASFV/POL/2015/Podlaskie |
| 166537 | Non-coding region | SNP | T- > C |  | MK628478 ASFV/LT14/1490, MH681419 ASFV/POL/2015/Podlaskie |
| 166555 | Non-coding region | SNP | T- > C |  | MK628478 ASFV/LT14/1490, MH681419 ASFV/POL/2015/Podlaskie |
| 166557 | Non-coding region | SNP | T- > C |  | MK628478 ASFV/LT14/1490, MH681419 ASFV/POL/2015/Podlaskie |
| 166841 | E199L | SNP | T- > C | Arg127 Gly | MK628478 ASFV/LT14/1490, MH681419 ASFV/POL/2015/Podlaskie |
| 166908 | E199L | SNP | T- > G | Gln104 His | LR713116 ASFV Georgia 2007/1, MK628478 ASFV/LT14/1490, MH681419 ASFV/POL/2015/Podlaskie |
| 173185 | Non-coding region | Indel | Deletion GGAATATATA |  | MK628478 ASFV/LT14/1490 |
| 182750 | I9R | SNP | G- > A | Glu90 Lys | MK628478 ASFV/LT14/1490, MH681419 ASFV/POL/2015/Podlaskie |
| 187827 | MGF 360-21R | SNP | C- > A | Pro24 His | MN393476 ASFV Wuhan 2019-1 |

**Supplementary Table S4.** List of SNPs and indels obtained from alignment of Arm/07/CBM/c2 with Arm/07/CBM/c4 using Nucmer. **[**Excel File].
